# Morphological and phylogenetic analyses revealed *Cystidichaete alba* gen. et sp. nov. (Phanerochaetaceae, Polyporales) from Southwest China

**DOI:** 10.3897/mycokeys.130.169753

**Published:** 2026-03-27

**Authors:** Xunchi Zhang, Daxiang Chen, Changlin Zhao

**Affiliations:** 1 Key Laboratory of Forest Disaster Warning and Control in Universities of Yunnan Province, Southwest Forestry University, Kunming 650224, China Department Microbial Drugs (MWIS), Helmholtz-Centre for Infection Research Braunschweig Germany; 2 College of Forestry, Southwest Forestry University, Kunming 650224, China College of Forestry, Southwest Forestry University Kunming China; 3 Yunnan Tongbiguan Provincial Nature Reserve, Mangshi 679319, China Key Laboratory of Forest Disaster Warning and Control in Universities of Yunnan Province, Southwest Forestry University Kunming China; 4 Modern Industry School of Edible-fungi, Southwest Forestry University, Kunming 650224, China Modern Industry School of Edible-fungi, Southwest Forestry University Kunming China; 5 Department Microbial Drugs (MWIS), Helmholtz-Centre for Infection Research, 38124 Braunschweig, Germany Yunnan Tongbiguan Provincial Nature Reserve Mangshi China

**Keywords:** Fungal classification, molecular systematics, new taxa, wood-inhabiting fungi

## Abstract

Wood-inhabiting fungi are a remarkably diverse group that play a fundamental role in ecosystem processes, particularly in wood degradation and the recycling of organic matter. As pivotal contributors to the intricate balance of forest ecosystems, these fungi are renowned as “key players” for their enzymatic prowess and products, which effectively breaks down woody components such as lignin, cellulose, and hemicelluloses. In the present study, a new wood-inhabiting fungal genus *Cystidichaete*, with its type species *C. alba*, collected from southwest China, is proposed based on a combination of morphological features and molecular evidence. The new genus *Cystidichaete* is characterized by the resupinate basidiomata with a smooth hymenophore, a monomitic hyphal system with clamped generative hyphae, abundant lamprocystidia, clavate basidia, and ellipsoid basidiospores. Sequences of the internal transcribed spacers (ITS), nuclear large subunit ribosomal RNA (nLSU), RNA polymerase second largest subunit (*rpb2*) and translation elongation factor 1-α (*tef1-α*) of the nuclear ribosomal DNA (rDNA) markers of the studied samples were generated. Phylogenetic analyses were performed using maximum likelihood, maximum parsimony, and Bayesian inference. Multi-locus phylogenetic analysis of ITS+nLSU+*rpb2*+*tef1-α* showed that *Cystidichaete* forms a monophyletic clade within the family Phanerochaetaceae (Polyporales), in which the new genus was grouped closely to *Stereophlebia* and *Gelatinofungus*.

## Introduction

Fungi are among Earth’s most diverse groups of organisms, playing indispensable roles in ecosystem processes and functioning (Hyde 2022). Wood-inhabiting fungi, primarily basidiomycetes, hold significant industrial, edible, medicinal, nutritional and other economic values (Dai 2010; Wu et al. 2019; Dai et al. 2021; Niego et al. 2023; Yuan et al. 2023; Dong et al. 2024). These fungi colonize diverse woody substrates, including living trees, standing deadwood, fallen logs, coarse branches, and stumps (M’Barek et al. 2020; Runnel et al. 2021; Dong et al. 2024). Wood-inhabiting fungi enzymatically degrade lignin, cellulose, and hemicelluloses through ligninolytic and cellulolytic enzymes, underscoring their critical role in organic matter recycling and nutrient cycling. These fungi are functionally categorized as white-rot or brown-rot taxa based on decay patterns (Bucher et al. 2004; Si et al. 2011; An et al. 2015; Liu et al. 2023).

Fungal classification within the kingdom Fungi is continually revised, driven by the integration of DNA sequence data into modern phylogenetic studies (Cui et al. 2019; Wijayawardene et al. 2020, 2022; Hyde et al. 2024; Dong et al. 2024). Given the early embrace of molecular systematics by mycologists, both the discovery and classification of fungi, among the more basal branches of the tree, are now emerging from genomic analyses and environmental DNA surveys (James et al. 2020).

Species of the family Phanerochaetaceae Jülich belong to wood inhabiting fungi, and they have industrial, medicinal, edible, and economic values while some others contain toxic metabolites (Chen et al. 2021; Dong et al. 2024; Xu et al. 2025). They produce ligninolytic and cellulolytic enzymes that break down lignin, cellulose, and hemicelluloses, underscoring their significance in the recycling of organic matter and nutrient cycling, typically categorized as the white-rot and brown-rot fungi (Cui et al. 2019; Chen et al. 2021).

The family Phanerochaetaceae belonging to the order Polyporales (Basidiomycota), was typified by *Phanerochaete* P. Karst. Twenty-eight genera were placed in this family Phanerochaetaceae as *Alboefibula* C.C. Chen & Sheng H. Wu, *Bjerkandera* P. Karst., *Callosus* C.L. Zhao, *Cremeoderma* Sheng H. Wu & C.C. Chen, *Crepatura* C.L. Zhao, *Donkia* Pilát, *Donkiella* J.H. Dong & C.L. Zhao, *Efibulella* Zmitr., *Gelatinofungus* Sheng H. Wu, et al., *Geliporus* Yuan Yuan, et al., *Hapalopilus* P. Karst., *Hyphodermella* J. Erikss. & Ryvarden, *Odontoefibula* C.C. Chen & Sheng H. Wu, *Oxychaete* Miettinen, *Paradonkia* Y. Xu & C.L. Zhao, *Neodonkiella* Y. Xu & C.L. Zhao, *Phaeophlebiopsis* Floudas & Hibbett, *Phanerina* Miettinen, *Phanerochaete* P. Karst., *Phlebiopsis* Jülich, *Pirex* Hjortstam & Ryvarden, *Porostereum* Pilát, *Quasiphlebia* C.C. Chen & Sheng H. Wu, *Rhizochaete* Gresl., Nakasone & Rajchenb., *Riopa* D.A. Reid, *Roseograndinia* Hjortstam & Ryvarden, *Stereophlebia* Zmitr. and *Terana* Adans according to recent studies (Dong et al. 2024; He et al. 2024; Larsson et al. 2025; Xu et al. 2025). Pioneering studies have been conducted on the phylogenetic analyses of Phanerochaetaceae, especially in *Phanerochaete* (Floudas and Hibbett 2015; Miettinen et al. 2016; Xu et al. 2020; Chen et al. 2021; Deng et al. 2024; Dong et al. 2024; Xu et al. 2025). The research using RPB1, RPB2, and the ITS and LSU ribosomal genes to investigate phanerochaetoid taxa and revealed that four clades of *Phanerochaete* sensu stricto, *Bjerkandera*, *Hyphodermella* and *Phlebiopsis* were divided, in which *Phanerochaete* s.s. and *Phlebiopsis* clades have been previously identified (Floudas and Hibbett 2015). Miettinen et al. (2016) carried on the phylogenetic relationships among genera within Phanerochaetaceae based on phylogenetic analyses of ITS, LSU and RPB1, which showed that the macromorphology of basidiomata and hymenophore construction did not reflect monophyletic groups in *Phanerochaete*. Further phylogeny of the five corticicoid genera of Phanerochaetaceae based on ITS1-5.8S-ITS2 and nrLSU sequence data showed that the *Donkia*, *Phlebiopsis*, *Rhizochaete*, and *Phanerochaete* clades formed a distinct lineage. Based on the ITS+nLSUrDNA gene regions, the research revealed that three species of *P. albocremea* Ying Xu & C.L. Zhao, *P. fissurata* Ying Xu & C.L. Zhao, and *P. punctata* Ying Xu & C.L. Zhao were located in the family *Phanerochaetaceae*, in which the species *P. fissurata* was retrieved as a sister to *P. cinerea* (Xu et al. 2025).

The corticioid species are the predominant morphological type in Phanerochaetaceae, but some have resupinated polypores and hydnaceous species (Chen et al. 2021; Zhao et al. 2024). The hyphal system of this family is usually monomitic, rarely dimitic, and the generative hyphae are usually simple septate, rarely nodose-septate, and cystidia are often present, and basidiospores are usually thin-walled, smooth, and colorless (Justo et al. 2017; Chen et al. 2021).

During investigations on wood-inhabiting fungi in the Yunnan-Guizhou Plateau, China, a large number of corticioid specimens were collected. To clarify the placement and relationships of these specimens, molecular phylogenetic along with taxonomic studies were carried out on the family Phanerochaetaceae based on the combined ITS+nLSU+*rpb2*+*tef1-α* data analysis. Accordingly, *Cystidichaete* gen. nov. (Phanerochaetaceae, Polyporales) and its novel species *C. alba* sp. nov. are formally described here, supported by morphological illustrations and multi-locus phylogeny.

## Materials and methods

### Sample collection and herbarium specimen preparation

The basidiomata were collected from fallen angiosperm branches in the Dehong Dai and Jingpo Autonomous Prefecture of Yunnan Province, Southwest China. The samples were photographed *in situ*, and fresh macroscopic details and other important information were recorded (Rathnayaka et al. 2025). Photographs were recorded by a Xiaomi 14 Ultra camera. Macroscopic observations were noted. Collected basidiomata were dried on an electric food dryer at 40 °C. Dried specimens were sealed in envelopes and zip-lock plastic bags and labeled with voucher numbers (Dong et al. 2025a). The voucher specimens were deposited in the herbarium of the Southwest Forestry University (SWFC), Kunming, Yunnan Province, P.R. China.

### Morphological study

The macro-morphological descriptions were based on field notes and photos taken in the field and in the lab. The color terminology follows Petersen (1996) and was confirmed in general terms according to the CMYK color code (Deep White Printing Team 2022). The micro-morphological data were obtained from dried specimens observed under a light microscope at 10 × 100 magnification (Zhao et al. 2023). Sections were mounted in 5% KOH and 2% Phloxine B (C_20_H_2_Br_4_C_l4_Na_2_O_5_) for microscopic observation. Cotton Blue and Melzer’s reagent were also used to examine micromorphological structures. Congo red was used as a stain when necessary (Horak 2005). To show the variation in spore sizes, 5% of measurements were excluded from each end of the range and shown in parentheses. At least 30 basidiospores from each specimen were measured. The following abbreviations are used: **KOH** = 5% potassium hydroxide water solution, **CB–** = acyanophilous, **IKI–** = both inamyloid and indextrinoid, **L** = mean spore length (arithmetic average for all spores), **W** = mean spore width (arithmetic average for all spores), **Q** = variation in the L/W ratios between the specimens studied, **Qm** represented the average Q of basidiospores measured ± standard deviation, and **n** = a/b (number of spores (a) measured from given number (b) of specimens).

### Molecular phylogenetic studies

The CTAB rapid plant genome extraction kit-DN14 (Aidlab Biotechnologies Co., Ltd, Beijing, China) was used to obtain genomic DNA from the dried fungal specimens according to the manufacturer’s instructions (Dong et al. 2024; Yang et al. 2025b). The extracted DNA was maintained at –20 °C for long-term storage. Four molecular markers were investigated, i.e., internal transcribed spacer (ITS), nuclear large subunit ribosomal RNA (nLSU), RNA polymerase II subunit 2 (*rpb2*) gene, and Translation elongation factor 1-α (*tef1-α*) gene and the primers and conditions are shown in Table 1. The PCR products were purified and sequenced at Kunming Tsingke Biological Technology Limited Company (Yunnan Province, China). All newly generated sequences were deposited in NCBI GenBank (https://www.ncbi.nlm.nih.gov/genbank/) (Table 2).

**Table 1.** Loci, primers, PCR amplification procedures, and references used in this study.

Name	Abbreviation	Name	Direction	Sequence (5'-3')	PCR amplification procedures	References
Internal transcribed spacer region of the rDNA	ITS	ITS5	Forward	GGAAGTAAAAGTCGTAACAAGG	94 °C 2 min; 35 cycles of 94 °C 60 s, 55 °C 60 s, 72 °C 2 min; 72 °C 10 min.	White et al. (1990)
		ITS4	Reverse	TCCTCCGCTTATTGATATGC		
Nuclear large subunit ribosomal	nLSU	LR0R	Forward	ACCCGCTGAACTTAAGC	94 °C 2 min; 35 cycles of 94 °C 30 s, 48 °C 1 min, 72 °C 1.5 min; 72 °C 10 min.	Vilgalys and Hester (1990)
		LR7	Reverse	TACTACCACCAAGATCT		
RNA polymerase second largest subunit	*rpb2*	RPB2-6F	Forward	TGGGGYATGGTNTGYCCYGC	94 °C 2 min; 9 cycles of 94 °C 45 s, 60 °C 45 s, 72 °C 1.5 min; 36 cycles of 94 °C 45 s, 53 °C 1 min, 72 °C 1.5 min; 72 °C 10 min.	Liu et al. (1999)
		RPB2-7cR	Reverse	CCCATRGCTTGYTTRCCCAT		
Translation elongation factor 1-α	*tef1-*α	EF1-983 F	Forward	GCYCCYGGHCAYCGTGAYTTYAT	94 °C 1 min; 35 cycles of 94 °C 30 s, 59 °C 1 min, 72 °C 1.5 min; 72 °C 10 min.	Rehner and Buckley (2005)
		EF1-2218R	Reverse	ATGACACCRACRGCRACRGTYTG		

**Table 2.** Names, voucher numbers, localities, references, and corresponding GenBank accession numbers of the taxa used in this study. New species are shown in bold; * refers to type material (holotype) and — refers to the missing data.

Species Name	Sample no.	GenBank Accession No.				Origin	References
		ITS	nLSU	*rpb2*	*tef1-α*		
*Alboefibula bambusicola*	Chen 2304*	MZ636926	MZ637091	OK135980	MZ913590	China	Chen et al. (2021)
*Alboefibula gracilis*	Wu 1809-106*	MZ636929	MZ637094	OK135982	MZ913591	China	Chen et al. (2021)
*Bjerkandera adusta*	HHB-12826-Sp	KP134983	KP135198	KP134913	KT305938	USA	Floudas and Hibbett (2015)
*Bjerkandera centroamericana*	L-13104-sp	KY948791	KY948855	—	—	Costa Rica	Justo et al. (2017)
*Callosus wenshanensis*	CLZhao 16017*	MW553934	MW553936	—	—	China	Chen et al. (2022)
*Callosus wenshanensis*	CLZhao 16034	MW553935	MW553937	—	—	China	Chen et al. (2022)
*Cremeoderma unicum*	Wu 1707-94	MZ636939	MZ637102	OK135987	MZ913705	China	Chen et al. (2021)
*Cremeoderma unicum*	Wu 1707-100	MZ636938	MZ637101	—	—	China	Chen et al. (2021)
*Crepatura ellipsospora*	CLZhao 1265*	MK343692	MK343696	—	—	China	Ma and Zhao (2019)
*Crepatura ellipsospora*	CLZhao 1260	MK343693	MK343697	—	—	China	Ma and Zhao (2019)
***Cystidichaete alba***	**CLZhao 39422**	**PX092380**	**PX092378**	**PX108328**	**PX108329**	China	**Present study**
***Cystidichaete alba***	CLZhao 39667*	**PX092381**	**PX092379**	**—**	**—**	China	**Present study**
*Donkia pulcherrima*	GC 1707-11	LC378994	LC379152	LC387351	LC387371	China	Chen et al. (2018)
*Donkia pulcherrima*	Gothenburg-2022	KX752591	KX752591	—	—	Austria	Miettinen et al. (2016)
*Donkiella yunnanensis*	CLZhao 3931*	OR094482	OR461467	OR733285	OR541925	China	Dong et al. (2024)
*Donkiella yunnanensis*	CLZhao 18292	OR094483	OR461468	OR733286	OR541926	China	Dong et al. (2024)
*Efibulella deflectens*	FCUG 1568	AF141619	AF141619	—	—	Sweden	Parmasto and Hallenberg (2000)
*Gelatinofungus brunneus*	GC 1703-31*	LC387339	LC387344	LC387367	LC387385	China	Chen et al. (2018)
*Gelatinofungus brunneus*	Wu 1207-162	MZ636978	MZ637139	OK136005	MZ913615	China	Chen et al. (2021)
*Gelatinofungus* sp.	RLG-10795-Sp	KY948785	KY948857	OK136007	MZ913619	USA	Justo et al. (2017)
*Geliporus exilisporus*	Dai 2172	KU598211	KU598216	—	—	China	Yuan et al. (2017)
*Geliporus exilisporus*	GC 1702-15	LC378995	LC379153	LC387352	LC387372	China	Chen et al. (2018)
*Hapalopilus percoctus*	H 7008581*	KX752597	KX752597	—	—	Botswana	Miettinen et al. (2016)
*Hapalopilus rutilans*	FP-102473-Sp	MZ636981	MZ637142	OK136004	MZ913723	USA	Chen et al. (2021)
*Hyphodermella corrugata*	MA-Fungi 24238	FN600378	JN939586	—	—	Portugal	Telleria et al. (2010)
*Hyphodermella rosae*	GC 1604-113	MZ636986	MZ637147	—	—	China	Chen et al. (2021)
*Hyphodermella rosae*	GC 1608-2	MZ636987	MZ637148	OK135983	MZ913592	Japan	Chen et al. (2021)
*Irpex lacteus*	FD-9	KP135026	KP135224	—	—	USA	Floudas and Hibbett (2015)
*Irpex latemarginatus*	FP-55521-T	KP135024	KP135202	KP134915	—	USA	Floudas and Hibbett (2015)
*Odontoefibula orientalis*	Wu 0910-57*	LC363490	LC363495	LC387362	LC387381	China	Chen et al. (2018)
*Odontoefibula orientalis*	GC 1703-76	LC379004	LC379156	LC387360	LC387379	China	Chen et al. (2018)
*Oxychaete cervinogilva*	GC 1501-16	MZ422783	MZ637173	—	MZ913613	China	Chen et al. (2021)
*Oxychaete cervinogilva*	Dmitry Schigel 5216	KX752596	KX752596	—	—	Australia	Chen et al. (2021)
*Paradonkia farinacea*	CLZhao 27184*	PQ527890	PQ527887	—	—	China	Xu et al. (2025)
*Paradonkia farinacea*	CLZhao 27221	PQ527891	PQ527888	—	—	China	Xu et al. (2025)
*Neodonkiella yinjiangensis*	CLZhao 30585*	PQ527892	PQ527889	—	—	China	Xu et al. (2025)
*Phaeophlebiopsis caribbeana*	HHB-6990	KP135415	KP135243	KP134931	MZ913643	USA	Floudas and Hibbett (2015)
*Phaeophlebiopsis peniophoroides*	FP-150577	KP135417	KP135273	—	—	USA	Floudas and Hibbett (2015)
*Phanerina mellea*	Wu 1010-34	MZ422784	MZ637176	—	—	China	Chen et al. (2021)
*Phanerina mellea*	WEI 17-224	LC387333	LC387340	LC387363	LC387382	China	Chen et al. (2018)
*Phanerochaete australis*	GC 1505-15	MZ422792	MZ637184	OK136010	MZ913595	China	Chen et al. (2021)
*Phanerochaete velutina*	GC 1604-56	MZ422844	MZ637224	OK136015	MZ913642	China	Chen et al. (2021)
*Phlebiopsis gigantea*	FCUG 1417	MZ637051	AF141634	OK135996	MZ913623	Norway	Liu et al. (2023)
*Phlebiopsis crassa*	GC 1602-45	MZ637049	MZ637251	OK135999	MZ913626	China	Chen et al. (2021)
*Pirex concentricus*	Kropp160Bup6-R	KP134985	—	—	—	USA	Floudas and Hibbett (2015)
*Pirex concentricus*	OSC-41587	KP134984	KP135275	KP134940	—	USA	Floudas and Hibbett (2015)
*Porostereum fulvum*	LY:18491	MG649452	MG649454	—	—	France	Chen et al. (2021)
*Porostereum spadiceum*	Wu 9508-139	MZ637062	MZ637263	OK136067	MZ913697	China	Chen et al. (2021)
*Quasiphlebia densa*	WEI 17-057	MZ637066	MZ637265	OK135986	MZ913630	China	Chen et al. (2021)
*Quasiphlebia densa*	Wu 9304-33	MZ637067	MZ637266	—	—	China	Chen et al. (2021)
*Rhizochaete filamentosa*	HHB-3169	KP135410	KP135278	KP134935	—	USA	Floudas and Hibbett (2015)
*Rhizochaete radicata*	FD-123	KP135407	KP135279	KP134937	—	USA	Floudas and Hibbett (2015)
*Riopa metamorphosa*	Spirin 2395	KX752601	KX752601	—	—	Russia	Miettinen et al. (2016)
*Riopa pudens*	Cui 3238	JX623931	JX644060	—	—	China	Jia et al. (2014)
*Roseograndinia jilinensis*	Wu 1307-137*	MZ637077	MZ637275	OK135985	MZ913632	China	Chen et al. (2021)
*Roseograndinia minispora*	WEI 18-508*	MZ637078	MZ637276	—	—	China	Chen et al. (2021)
*Stereophlebia pendula*	GB:KHL 15159	PQ013066	PQ013066	—	—	Spain	Larsson et al. (2025)
*Stereophlebia pendula*	GB:EL 29-11	PQ013067	PQ013067	—	—	Spain	Larsson et al. (2025)
*Terana caerulea*	FP-104073	KP134980	KP135276	KP134960	—	USA	Floudas and Hibbett (2015)
*Terana caerulea*	GC 1507-2	MZ637090	MZ637287	OK136037	MZ913654	China	Chen et al. (2021)

**Figure 1.**
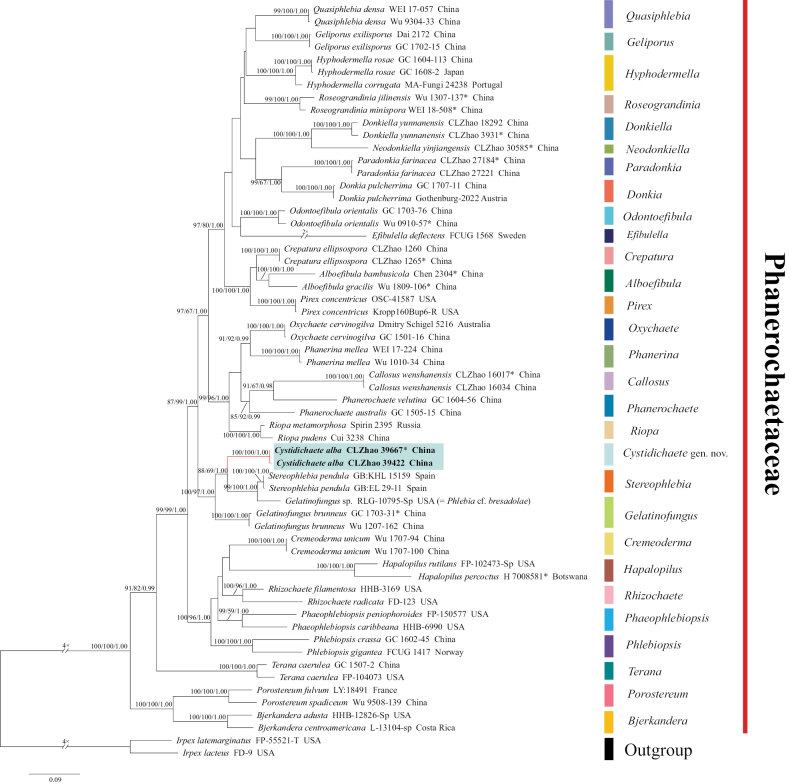
Maximum Likelihood analysis illustrating the phylogeny of *Cystidichaete* and related genera in the family Phanerochaetaceae based on ITS+nLSU+*rpb2*+*tef1-α* sequences. Branches are labelled with maximum likelihood bootstrap value ≥ 70%, parsimony bootstrap value ≥ 50%, and Bayesian posterior probabilities ≥ 0.95. Colored bars represent different genera. New species are shown in bold, * refers to type material (holotype).

Sequences generated for this study were aligned, with additional sequences downloaded from GenBank. Sequences were aligned in MAFFT 7 (https://mafft.cbrc.jp/alignment/server/) adjusting the direction of nucleotide sequences according to the first sequence (accurate enough for most cases), and selecting the G-INS-i iterative refinement method (Katoh et al. 2019). The alignment was adjusted manually using AliView version 1.27 (Larsson 2014). The dataset was first aligned, and then the sequences of ITS+nLSU+*rpb*2+ *tef*1-α were combined in Mesquite v. 3.81. The combined ITS+nLSU+*rpb2*+ *tef1-α* dataset was used to infer the phylogenetic analysis of the new genus and related species within the Phanerochaetaceae. For this analysis, *Irpex lacteus* (Fr.) Fr. *and Irpex latemarginatus* (Durieu & Mont.) C.C. Chen & Sheng H. Wu served as the outgroup to root the tree (Fig. 1; Dong et al. 2024). The alignment datasets were deposited in TreeBASE (Submission ID 32269).

Maximum Parsimony (MP), Maximum Likelihood (ML), and Bayesian Inference (BI) analyses were applied to the combined three datasets following previous studies (Zhao and Wu 2017; Yang et al. 2025a). Maximum Parsimony (MP) analysis was performed in PAUP* v. 4.0b10 (Swofford 2002). All characters were equally weighted, and gaps were treated as missing data. Trees were inferred using the heuristic search option with TBR branch swapping and 1000 random sequence additions. Max trees were set to 5000, branches of zero length were collapsed, and all parsimonious trees were saved. Clade robustness was assessed using bootstrap (BT) analysis with 1000 replicates (Felsenstein 1985). Descriptive tree statistics, tree length (TL), consistency index (CI), retention index (RI), rescaled consistency index (RC), and homoplasy index (HI) were calculated for each maximum parsimonious tree generated. Maximum likelihood (ML) analysis was performed with RAxML-HPC BlackBox in CIPRES Science Gateway (https://www.phylo.org/portal2/login!input.action, Miller et al. 2012) using a GTRCAT model of evolution with 1,000 bootstrap replicates. ModelFinder v2.2.0 (Kalyaanamoorthy et al. 2017) was used to select the best-fit model using Bayesian Information Criterion (BIC). Bayesian Inference (BI) phylogenies by PhyloSuite v1.2.3 (Zhang et al. 2020; Xiang et al. 2023) were inferred using MrBayes v3.2.7a (Ronquist et al. 2012). Branches were considered significantly supported if they received a Maximum Likelihood bootstrap value (BS) of ≥ 70%, a Maximum Parsimony bootstrap value (BT) of ≥ 50%, or Bayesian Posterior Probabilities (BPP) of ≥ 0.95.

## Results

### Phylogenetic analyses

The combined ITS+nLSU+*rpb2*+ *tef1-α* dataset (Fig. 1) included sequences from 60 fungal specimens representing 43 species. The dataset had an aligned length of 3987 characters, of which 2504 were constant, 316 were variable and parsimony-uninformative, and 1167 were parsimony-informative. Maximum Parsimony (MP) analysis yielded one equally parsimonious tree (TL = 6874, CI = 0.3667, HI = 0.6333, RI = 0.4944, and RC = 0.1813).

The best RAxML tree with a final likelihood value of -33260.831640 is presented. The evolutionary model GTR+GAMMA is applied to all the genes. The matrix contained 1762 distinct alignment patterns, with 38.82% of characters undetermined (gaps). Estimated base frequencies were as follows: A = 0.243058, C = 0.238649, G = 0.272340, T = 0.245954; substitution rates AC = 0.884598, AG = 2.826789, AT = 1.650582, CG = 0.888040, CT = 5.769647, GT = 1.000000; gamma distribution shape parameter α = 0.217174.

The best-fit model for the dataset according to BIC: GTR+I+G4. Bayesian analysis was performed under the GTR+I+G4 model (2 parallel runs, 1000000 generations), in which the initial 25% of sampled data were discarded as burn-in. Both Bayesian Inference (BI) and Maximum Parsimony (MP) analyses resulted in a similar topology to the Maximum Likelihood (ML) analysis, with an average standard deviation of split frequencies = 0.007990 (BI), and the effective sample size (ESS) for Bayesian analysis across the two runs is the double of the average ESS (avg. ESS) = 579.5.

Based on separate analyses of ITS, nLSU, *rpb2*, and *tef1-α* sequences, NCBI BLAST searches performed on 25 August 2025 returned the top ten similar taxa with significant alignments (Table 3).

**Table 3.** Summary of NCBI BLAST results for *Cystidichaete alba* based on ITS, nLSU, *rpb2*, and *tef1-α* sequences (accessed on 25 August 2025).

Gene Locus	Top Ten Similar Taxa with Significant Alignments	Maximum Record Descriptions					References
		Max Score	Total Score	Query Cover	E value	Identity	
ITS	*Stereophlebia pendula* (Fr.) K.H. Larss.	1005	1005	97%	0	95.71%	Larsson et al. (2025)
nLSU	*Stereophlebia pendula* (Fr.) K.H. Larss.	2438	2438	98%	0	99.04%	Larsson et al. (2025)
	*Gelatinofungus betulina* Shun Liu & Biao Zhu						Liu et al. (2025)
	*Gelatinofungus* sp. strain RLG-10795-Sp						Justo et al. (2017)
	*Phlebiopsis daweishanensis* J.H. Dong & C.L. Zhao						Dong et al. (2024)
	*Phlebiopsis xuefengensis* J. Zou						Li et al. (2021)
	*Riopa pudens* Miettinen						Dai YC lab submission
	*Phlebiopsis pilatii* (Parmasto) Spirin & Miettinen						Miettinen et al. (2016)
	*Ceriporia mellea* (Berk. & Broome) Ryvarden						Jia et al. (2014)
*rpb2*	*Gelatinofungus* sp. strain RLG-10795-Sp	651	651	100%	0	82.64%	Chen et al. (2021)
	*Gelatinofungus brunneus* Sheng H. Wu, C.C. Chen & C.L. Wei						Chen et al. (2021)
	*Phanerochaete ericina* (Bourdot) J. Erikss. & Ryvarden						Floudas and Hibbett (2015)
	*Phanerochaete thailandica* Kout & Sádlíková						Chen et al. (2021)
	*Phlebiopsis alpina* C.C. Chen, Sheng H. Wu & S.H. He						Chen et al. (2021)
	*Ceriporia camaresiana* (Bourdot & Galzin) Bondartsev & Singer						Větrovský et al. (2016)
*tef1-α*	*Gelatinofungus brunneus* Sheng H. Wu, C.C. Chen & C.L. Wei	1476	1476	100%	0	92.82%	Chen et al. (2021)
	*Gelatinofungus* sp. strain RLG-10795-Sp						Justo et al. (2017)
	*Terana caerulea* (Lam.) Kuntze						Chen et al. (2021)
	*Peniophora nuda* (Fr.) Bres., Plicaturopsis crispa (Pers.) D.A. Reid						Brandon Matheny et al. (2007)
	*Panaeolina foenisecii* (Pers.) Maire						Direct Submission

The topology based on ITS+nLSU+rpb2+ tef1-α sequences (Fig. 1) showed that the new taxon was clustered into Phanerochaetaceae, with which it formed a distinct lineage, and sister to *Stereophlebia* and *Gelatinofungus* with 100% BS, 97% BP, 1.00 BPP support. Accordingly, we introduce *Cystidichaete* gen. nov., with *Cystidichaete alba* sp. nov. designated as the type species.

### Taxonomy

#### Phanerochaetaceae

Taxon classificationFungiPolyporalesPhanerochaetaceae

Jülich

CB1EC99D-8C14-5091-A6D0-B2AA789BAA90

##### Type genus.

*Phanerochaete* P. Karst.

##### Description.

Mostly corticioid species, along with a few resupinate or pileate polypores, and pileate hydnaceous species; hyphal system usually monomitic, rarely dimitic; hyphae usually simple septate, rarely nodose-septate; basidiospores thin-walled, smooth, colorless; cystidia often present. Producing a white rot. (Chen et al. 2021).

##### Accepted genera.

*Alboefibula*, *Bjerkandera*, *Callosus*, *Cremeoderma*, *Crepatura*, *Cystidichaete*, *Donkia*, *Donkiella*, *Efibulella*, *Gelatinofungus*, *Geliporus*, *Hapalopilus*, *Hyphodermella*, *Odontoefibula*, *Oxychaete*, *Paradonkia*, *Neodonkiella*, *Phanerina*, *Phanerochaete*, *Phaeophlebiopsis*, *Phlebiopsis*, *Pirex*, *Porostereum*, *Quasiphlebia*, *Rhizochaete*, *Riopa*, *Roseograndinia*, *Stereophlebia* and *Terana*.

##### Notes.

Phanerochaetaceae was established by Jülich (1982) with the genus *Phanerochaete* as the type genus. It belongs to the phlebioid clade within the order Polyporales and causes a white rot (Larsson 2007; Binder et al. 2013; Miettinen et al. 2016; Justo et al. 2017). In the current study, 29 genera are accepted in Phanerochaetaceae, *Cystidichaete* gen. nov., which is newly proposed in this study.

#### Cystidichaete

Taxon classificationFungiPolyporalesPhanerochaetaceae

X.C. Zhang & C.L. Zhao gen. nov.

024D9E4B-25D4-50A3-9AFC-A9A3ED05864E

860430

##### Chinese name.

囊状体革菌属 (nang zhuang ti ge jun shu).

##### Etymology.

*Cystidichaete* (Lat.): refers to the abundant cystidia in the hymenium.

##### Description.

Basidiomata annual, resupinate, adnate, membranaceous, without odor or taste when fresh, becoming fragile upon drying. Hymenial surface smooth, white when fresh, white to cream when dry. Hyphal system monomitic; generative hyphae with clamp connections, colorless, thin- to slightly thick-walled. Subicular hyphae thin- to slightly thick-walled. Lamprocystidia abundant, arising from subhymenium, subulate, heavily encrusted with crystals, distinctly thick-walled, embedded in subhymenium or exerted. Basidia clavate, occasionally with oil drops, with four sterigmata and a basal clamp connection. Basidiospores ellipsoid, colorless, thin-walled, smooth, occasionally with oil drops, IKI–, CB–. Causing a white rot.

##### Type species.

*Cystidichaete alba* X.C. Zhang & C.L. Zhao.

##### Notes.

In our analyses, *Cystidichaete* is formed a distinct group typified by *C. alba*. The new genus placed within Phanerochaetaceae (Polyporales) and grouped with *Stereophlebia* and *Gelatinofungus* with 100% BS, 97% BP, 1.00 BPP support (Fig. 1). However, morphologically, *Stereophlebia* differs from *Cystidichaete* by having chondrostereoid to phlebioid basidiomata, prostrate to reflexed with differentiated upper tomentum, a smooth or irregularly costate to tuberculate hymenophore, and lacking lamprocystidia (Zmitrovich 2018). *Gelatinofungus* differs from *Cystidichaete* by having ceraceous basidiomata, a dark purplish gray to cinnamon-brown hymenial surface that darkens in KOH, a smooth to tuberculate hymenophore, and lacking lamprocystidia (Chen et al. 2021).

#### Cystidichaete alba

Taxon classificationFungiPolyporalesPhanerochaetaceae

X.C. Zhang & C.L. Zhao sp. nov.

44E379D1-C10C-59E1-A3CB-2E753E292F4F

860431

Figs 2, 3, 4

##### Chinese name.

白囊状体革菌 (bai nang zhuang ti ge jun).

**Figure 2.**
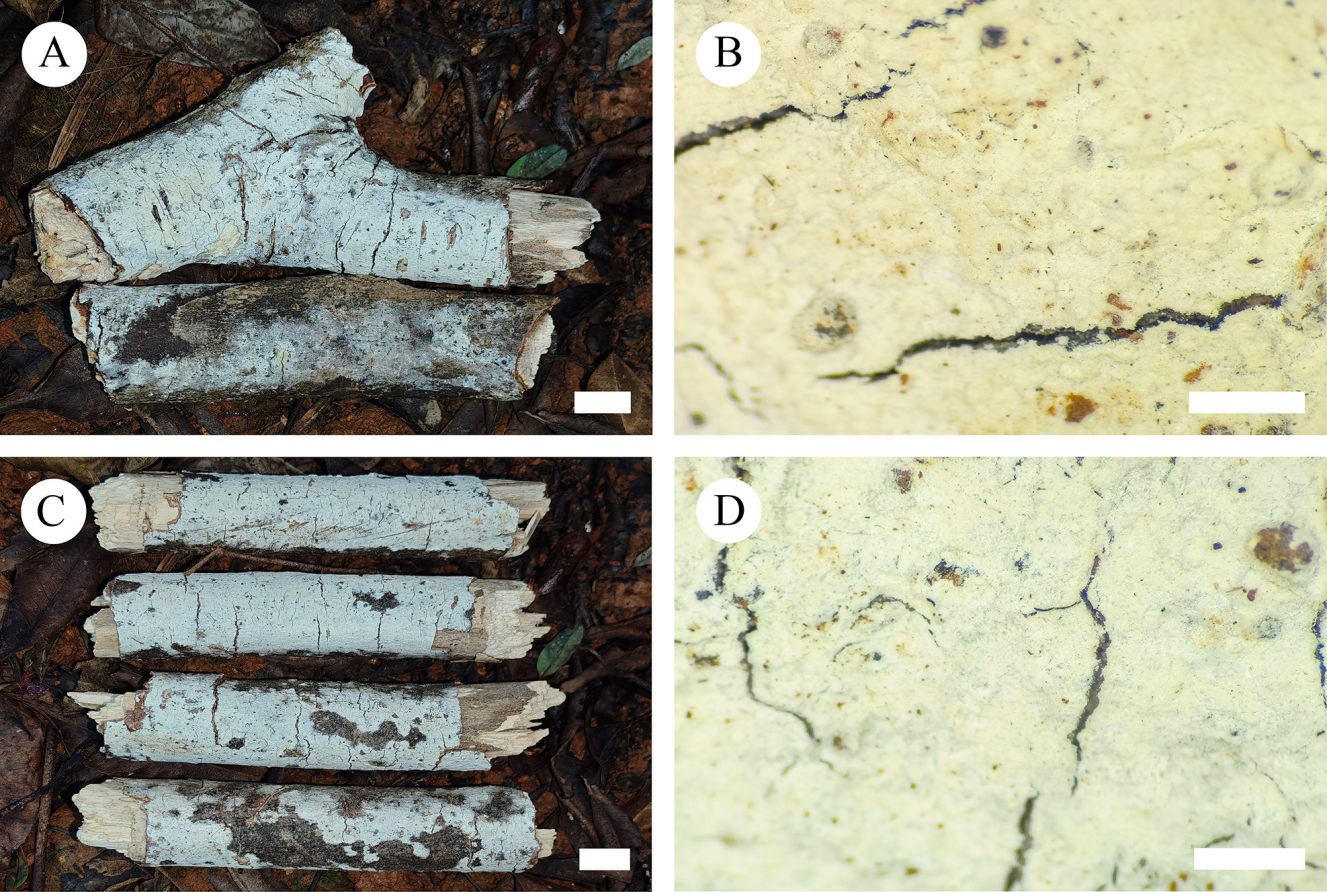
Basidiomata of *Cystidichaete alba*, in general and in detail. **A, B**. CLZhao 39422; **C, D**. CLZhao 39667 (holotype). Scale bars: 1 cm (**A, C**); 1 mm (**B, D**).

**Figure 3.**
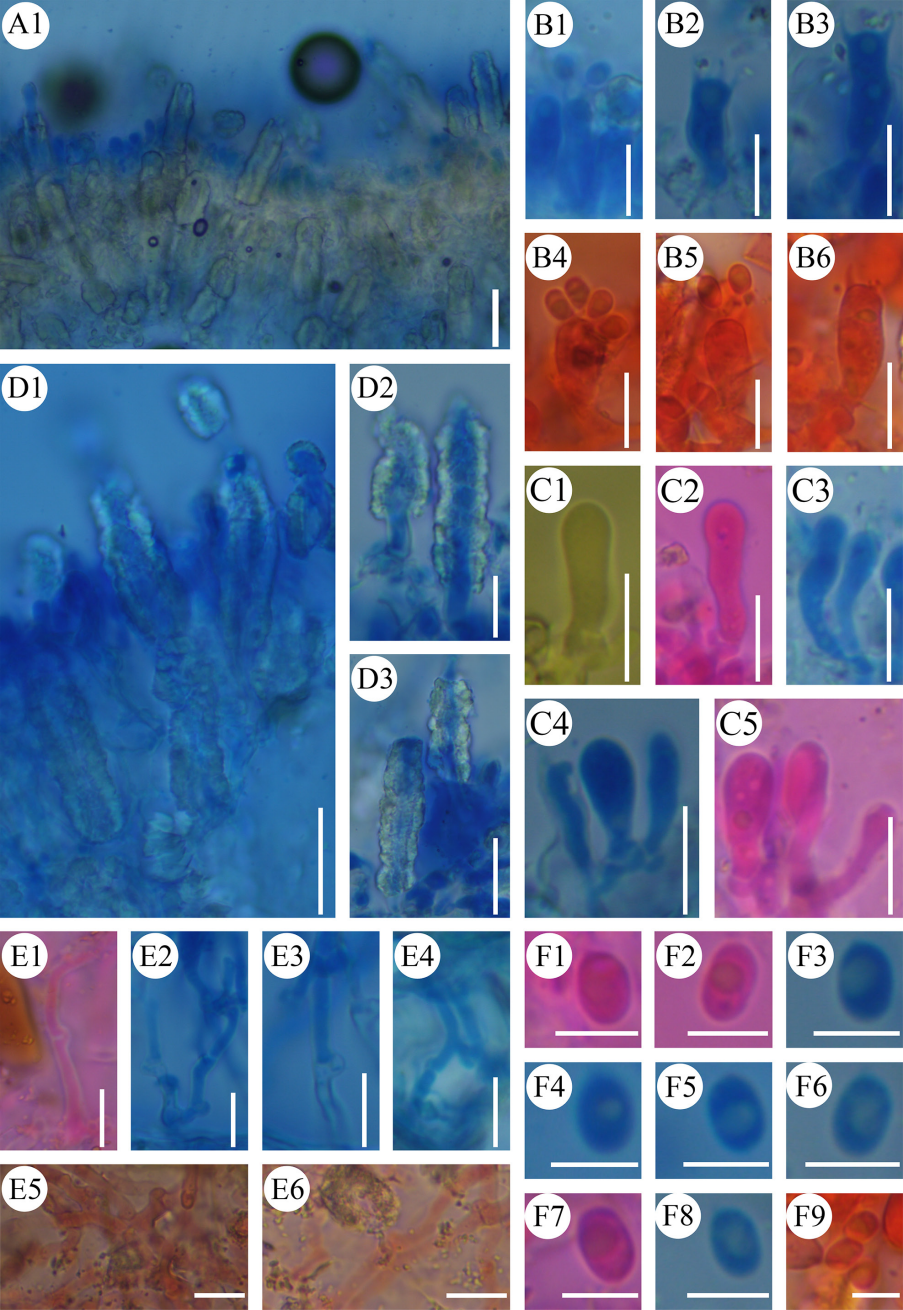
Microscopic structures of *Cystidichaete alba* (CLZhao 39667, holotype). **A1**. A section of the hymenium; **B1–B6**. Basidia; **C1–C5**. Basidioles; **D1–D3**. Lamprocystidia; **E1–E4**. Subicular hyphae; **E5, E6**. Hyphae; **F1–F9**. Basidiospores. Scale bars: 20 µm (**A1**); 10 µm (**B1–B6**) 10 µm (**C1–C5**); 20 µm (**D1, D3**); 10 µm (**D2**); 10 µm (**E1–E6**); 5µm (**F1–F9**).

**Figure 4.**
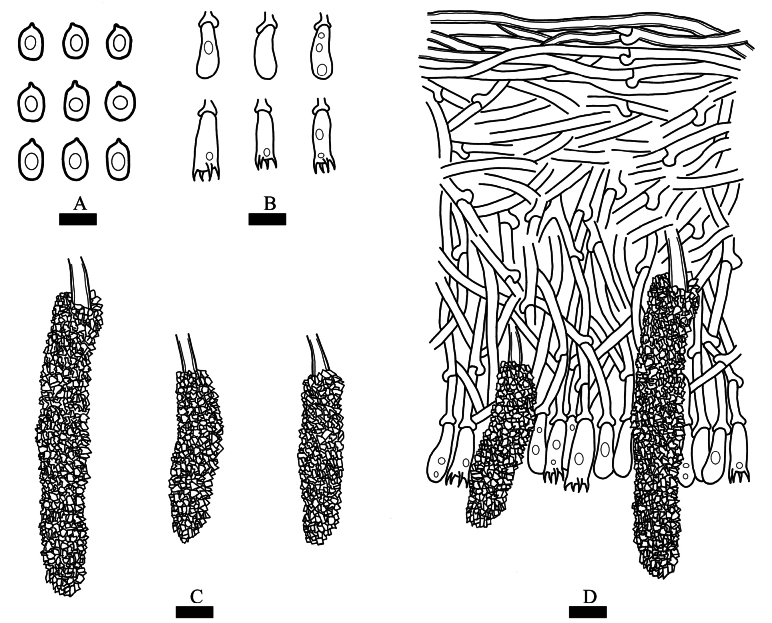
Microscopic structures of *Cystidichaete alba* (Drawn from the holotype CLZhao 39667). **A**. Basidiospores; **B**. Basidia and basidioles; **C**. Lamprocystidia; **D**. Section through basidioma. Scale bars: 5 µm (**A**); 10 µm (**B–D**).

##### Holotype.

China • Yunnan Province, Dehong Dai and Jingpo Autonomous Prefecture, Mangshi, Tongbiguan Nature Reserve, GPS coordinates 24°22'N, 97°50'E, altitude 1405 m asl., on fallen angiosperm branch, leg. C.L. Zhao, 8 July 2024, CLZhao 39667 (SWFC 00039667).

##### Etymology.

alba (Lat.): refers to the species having a white hymenial surface.

##### Basidiomata.

Annual, resupinate, closely adnate, membranaceous, without odor or taste when fresh, becoming fragile upon drying, up to 8 cm long, 2 cm wide, and 160 µm thick. Hymenial surface smooth, white (C0 M0 Y5 K0) when fresh, white (C0 M0 Y5 K0) to cream (C0 M5 Y30 K0) upon drying. Sterile margin grayish-white (C0 M0 Y0 K5), narrow, up to 2 mm wide.

##### Hyphal structure.

Monomitic, generative hyphae with clamp connections, colorless, thin- to slightly thick-walled, branched, interwoven, 2–3.5 µm in diameter; ubicular hyphae thin- to slightly thick-walled 3–3.5 µm in diameter. IKI–, CB–; tissues unchanged in KOH.

##### Hymenium.

Lamprocystidia abundant, arising from subhymenium, subulate, heavily encrusted with crystals, distinctly thick-walled, embedded in subhymenium or exerted, 35–68 × 8–15 µm. Basidia clavate, occasionally with oil drops, with four sterigmata and a basal clamp connection, 13–20× 4–6 µm; basidioles dominant, occasionally with oil drops, similar to basidia in shape, but slightly smaller.

##### Basidiospores.

Ellipsoid, colorless, smooth, thin-walled, often with oily drops, IKI–, CB–, (4–)4.4–5.8(–6) × (2–)2.5–3.8(–4) µm, L = 5.10 µm, W = 3.20 µm, Q = 1.59, Qm = 1.59 ± 0.05 (n = 30/1).

##### Type of rot.

White rot.

##### Additional specimen (paratype) examined.

China • Yunnan Province, Dehong Dai and Jingpo Autonomous Prefecture, Mangshi, Tongbiguan Nature Reserve, GPS coordinates 24°22'N, 97°50'E, altitude 1405 m asl., on fallen angiosperm branch, leg. C.L. Zhao, 8 July 2024, CLZhao 39422 (SWFC 00039422).

## Discussion

The Dehong Dai and Jingpo Autonomous Prefecture, located in Southwestern China, is renowned as one of the most biologically diverse regions in the country. Its topography and diverse ecosystems, making it a focal point for fungal biodiversity in China. Recently, studies on fungal diversity and the ecology of Basidiomycota in Dehong, Yunnan Province, were conducted (Chen et al. 2025; Deng et al. 2025; Gu et al. 2025; Liu et al. 2025a, 2025b; Xu et al. 2025; Yang et al. 2025b). Integrated scientific research indicated that Tongbiguan Nature Reserve features tall trees and vertically zoned vegetation succession, encompassing tropical monsoon forests, tropical montane rainforests, south-subtropical monsoon evergreen broad-leaved forests, mid-mountain humid evergreen broad-leaved forests, bamboo forests, shrublands, and grasslands, in which this heterogeneous habitat fosters exceptional macrofungal diversity (Yang and Du 2006).

The generic-level phylogeny of Phanerochaetaceae has become increasingly well resolved, with several new genera erected to accommodate independent lineages (Floudas and Hibbett 2015; Miettinen et al. 2016; Yuan et al. 2017; Chen et al. 2018, 2021; Ma and Zhao 2019; Dong et al. 2024, 2025b; Luo et al. 2024; Xu et al. 2025). At present, 29 genera are accepted in the family, most of which are corticioid fungi (Chen et al. 2021; Xu et al. 2025). Although most of the newly described taxa in the family originated from East Asia, several lineages in this region still require further systematic study based on more comprehensive sampling. Our multilocus phylogenetic analyses using combined ITS+nLSU+*rpb2*+*tef1-α* sequences analysis (Fig. 1), placed the new genus and its novel species within Phanerochaetaceae (Polyporales, Basidiomycota).

Phylogenetically, the phylogram based on the combined ITS+nLSU+*rpb2*+*tef1-α* dataset showed that *Cystidichaete alba* clustered with *Stereophlebia pendula* (Fr.) K.H. Larss and *Gelatinofungus brunneus* Sheng H. Wu, C.C. Chen & C.L. Wei with strong support (100% BS, 97% BP, 1.00 BPP; Fig. 1). However, *Stereophlebia pendula* differs from *Cystidichaete alba* by its effused to effused reflexed, membranous basidiomata, hymenium smooth to tuberculate, sometimes with radiating ridges near the margin, cream to ochraceous to pale orange reddish, pileus surface smooth with adpressed hyphae and longer basidiospores (5.7–7.1 × 2.7–3.3 µm vs. 4.4–5.8 × 2.5–3.8 µm; Larsson et al. 2025). *Gelatinofungus brunneus* differs from *Cystidichaete alba* by its ceraceous basidiomata, hymenial surface dark purplish gray with brownish tints when young, becoming cinnamon to brown when old, darkening in KOH, tuberculate, uncracked and longer basidia (20–38 × 5–6 µm vs. 13–20× 4–6 µm) (Chen et al. 2021).

Phanerochaetaceae is a major group of wood-inhabiting fungi (Basidiomycota) characterized by relatively simple basidiomata and fewer diagnostic morphological features than those of polypores and mushrooms (Xu et al. 2020). Despite this morphological simplicity, they exhibit higher species and phylogenetic diversity, yet remain substantially understudied (Larsson et al. 2004; Binder et al. 2005; Bernicchia and Gorjón 2010; Dai 2011; Sun et al. 2020). A substantial number of Phanerochaetaceae remain undocumented worldwide, particularly in subtropical and tropical ecosystems (Dong et al. 2024; Zhou et al. 2024; Yang et al. 2025b). As shown in this study and earlier ones (Volobuev et al. 2015; Chen et al. 2018; Xu et al. 2025), DNA sequence data are invaluable for resolving cryptic diversity and delimiting taxa within Phanerochaetaceae. Therefore, future taxonomic studies should continue to integrate detailed morphological assessments with multilocus phylogenetic analyses to better document species diversity and to refine our understanding of the phylogeny and evolutionary history of the family.

## Supplementary Material

XML Treatment for Phanerochaetaceae

XML Treatment for Cystidichaete

XML Treatment for Cystidichaete alba

## Additional information

### Use of AI

No use of AI was reported.

### Author ORCIDs

Xunchi Zhang https://orcid.org/0000-0003-3887-0979

Daxiang Chen https://orcid.org/0009-0001-2309-1991

Changlin Zhao https://orcid.org/0000-0002-8668-1075

### Data availability

All of the data that support the findings of this study are available in the main text.
